# Effects of Smoking on Inflammatory-Related Cytokine Levels in Human Serum

**DOI:** 10.3390/molecules27123715

**Published:** 2022-06-09

**Authors:** Hongjuan Wang, Huan Chen, Yaning Fu, Min Liu, Jingni Zhang, Shulei Han, Yushan Tian, Hongwei Hou, Qingyuan Hu

**Affiliations:** China National Tobacco Quality Supervision and Test Center, Key Laboratory of Tobacco Biological Effects, Zhengzhou, No. 6, Cuizhu Street, Gaoxin District, Zhengzhou 450001, China; redbri2013@163.com (H.W.); hunny_ch@163.com (H.C.); fynmail@126.com (Y.F.); ggayt0616@163.com (M.L.); zhangjnlife@163.com (J.Z.); hsl1983@163.com (S.H.); yushantian@126.com (Y.T.)

**Keywords:** smoking, inflammatory, cytokine, human serum

## Abstract

Cardiovascular and respiratory diseases, and several cancers resulting from tobacco smoking, are initially characterized by chronic systemic inflammation. Cytokine imbalances can result in inflammation, making it important to understand the pathological mechanisms behind cytokine production. In this study, we collected blood samples from 78 healthy male volunteers, including non-smokers (*n* = 30), current smokers (*n* = 30), and ex-smokers (*n* = 18), and utilized the liquid suspension chip technique to investigate and compare the expression levels of 17 cytokines and chemokines in the human serum of these volunteers. The results demonstrated that the expression levels of CXCL9/MIG and sIL-6R significantly increased after smoking, and continued to increase after quitting smoking. The expression levels of TARC, ITAC, and sVEGFR-3 increased after smoking but decreased after quitting smoking; the expression level of SAA significantly decreased after smoking and showed an upward trend after quitting smoking. Seven cytokines (IL-1β, BCA-1, TNF-α, CRP, ENA-78, MDC, and TNFRII) did not vary between the three groups, while four cytokines (IL-1α, IL-6, IL-8, and SCF) were not detected in any serum sample. In conclusion, this study assessed the physiological production of cytokines and chemokines, highlighting the differences in each due to smoking status. Our results could help evaluate the early development of smoking-related chronic diseases and cancers.

## 1. Introduction

Inflammatory responses are driven by a complex network of mediators and signaling pathways. For example, cytokines regulating inflammatory responses include interleukins, whose core function is the orchestration of different cells of the immune system during host defense against pathogens, chemokines that promote chemotaxis, and interferons. Moreover, these molecules are involved in innate and adaptive immunity, and play a significant physiological role in lymphoid tissue ontogenesis, organogenesis, vasculogenesis, and tissue repair [1]. Diseases often occur when the expression of these molecules is chronically altered. Indeed, chronic inflammatory diseases are recognized as the most significant cause of death globally, with more than 50% of all deaths attributable to inflammation-related diseases [2]. Treating inflammatory diseases poses a significant challenge to medical science. Environmental factors play important roles in the development and progression of inflammatory-related diseases, including tobacco smoking, which can upset the homeostasis of the immune system by modulating immune-regulatory activities, leading to inflammation [3,4,5].

Despite widespread knowledge of the health risks associated with smoking, tobacco use remains high in developing countries [6]. Worldwide, tobacco smoking is reported to have killed almost 6 million people annually, including approximately 600,000 non-smokers who died from second-hand smoke exposure [7,8,9]. Therefore, tobacco smoking is a threat to public health, and a worldwide epidemic [10].

There are several ways that tobacco smoking can lead to inflammatory and autoimmune diseases, including genetic/epigenetic modifications, increased oxidative stress, and free radical production [11,12,13,14]. These effects can increase the proliferation of B and T cells; reduce the generation, activity, and autoantibody of immuno-suppressive T regulatory (Treg) cells; enhance the expression of pro-inflammatory mediators, such as Interleukin-1 beta (IL-1β), Interleukin-6 (IL-6), Interleukin-8 (IL-8), and tumor necrosis factors (TNFs); and enhance the expression of chemotactic cytokines, such as recombinant human C-X-C Motif Chemokine Ligand 9 (CXCL9/MIG), thymus and activation-regulated chemokine (TARC), and IFN-inducible T cell α chemoattractant (ITAC) [15,16,17,18,19,20,21,22,23]. Chemokines are small-molecule secretory proteins that can mediate cell directional migration, activate cellular immune activity, and participate in immune regulation [24]. CXCL9 is a member of the CXC chemokine family, and plays an important role in the chemotaxis of immune cells. T lymphocytes can be recruited to inflammatory sites through CXCL9 chemotaxis, enhancing the proliferation of T lymphocytes and the production of cytokines in allogeneic reaction [25,26]. Studies have found that CXCL9 plays an important role in many diseases, including external infection, autoimmune diseases, tumor treatment, lymphoma [27], and fatty livers [28]. Changes in these inflammatory markers and cytokines can lead to cancers in 18 different tumor sites and a range of other chronic diseases, including coronary heart disease, stroke, and chronic obstructive pulmonary disease [29,30]. Exposure to tobacco smoke can also regulate the expression of cytokine receptors [31]. IL-6 is involved in many biological processes, such as inflammation and immune regulation, while the imbalance of immune regulation of the IL-6/ IL-6 receptor axis can lead to various inflammatory diseases, such as rheumatoid arthritis and chronic hepatitis [32]. After specifically binding with sIL-6R, it can activate the downstream classical signal pathway, such as the JAK/STAT3 and PI3K/Akt signaling pathways; it can then activate T cells to mediate the secretion of inflammatory factors by immune cells such as neutrophils, fibroblasts, and macrophages, and promote the occurrence of inflammation [33,34]. These interleukins, chemokines, and other inflammatory-related cytokines play an important role in transmitting information, activating and regulating immune cells, and mediating inflammatory responses, all of which are closely related to tobacco smoke intake. Most previous studies have described the association between tobacco smoking and diseases. For instance, smoking is known to affect the concentration and activation of some white blood cells, including leukocytes, which are associated with increased concentrations of the inflammatory markers C-reactive protein (CRP) and IL-6 [35]. However, the changes to these cytokines in the serum after smoking cessation requires further study. ITAC is distributed in a small amount in the lung, pancreas, spleen, and other organs or tissues of normal individuals, mainly expressed by bronchial epithelial cells and vascular endothelial cells, which mostly induce the directional migration of T cells and trigger an inflammatory response; inhibit the growth of vascular endothelial cells; and regulate angiogenesis, among other biological functions [36]. Soluble vascular endothelial growth factor receptor 3 (sVEGFR-3) is a novel receptor that regulates lymphangiogenesis by inhibiting vascular endothelial growth factor (VEGF)-C and making it unable to activate cognate receptors; it is a potential biomarker for the antiangiogenic activity of tyrosine kinase inhibitors [37,38,39]. Serum amyloid A (SAA)is a pro-inflammatory factor, because it is an effective chemokine that mediates leukocyte migration and stimulates the expression of pro-inflammatory mediators in vitro and in vivo. Since SAA is common in chronic obstructive pulmonary disease (COPD) and lung cancer, it can predict the severity of these two diseases, and has been identified as a biomarker of lung cancer severity and a potential target for treatment [40,41,42].

Smoking causes 140,000 premature deaths from cardiovascular disease (CVD) annually in the United States, representing approximately 30% of all smoking-related deaths [43]. Inflammation is one mechanism by which cigarette smoking could affect CVD [44,45]. A growing body of evidence outlines the relationship between individual inflammatory markers and smoking status. While smoking can cause immune-related diseases, the mechanism behind how it affects inflammation is poorly understood. To understand the changes to typical inflammatory cytokines with smoking and cessation of smoking, and to explore smoking-related inflammation, we measured 17 inflammatory cytokines in the serum of non-smokers, ex-smokers, and current smokers. Bio-plex suspension chip technology was used to investigate the changes in the 17 inflammatory cytokines in the serum of these subjects. This study was designed to provide insights into potential health risks related to the expression of cytokines and chemokines in human serum, based on smoking status.

## 2. Materials and Methods

### 2.1. Study Design and Participants

This study was approved by the Life Science Ethics Committee of Zhengzhou University. Healthy male non-smokers, and smokers between 18 and 60 years of age, were recruited for this study, while any subject with the following conditions was excluded: an acute or chronic infectious disease; a clinically significant disorder; or medicating with drugs known to influence immunological factors (e.g., corticosteroids). All subjects lived in a typical rural area in central China and had similar occupations, lifestyles, and environmental exposure factors. There was no significant difference between or within these groups. Once informed consent was obtained, each participant completed a self-evaluation questionnaire assessing their history of cigarette smoking. According to relevant WHO standards, participants were categorized by smoking status as follows: “non-smoker” (total lifetime smoking <100 cigarettes, 30 males); “current smoker” (≥5 cigarettes/day; smoking time ≥6 months; 30 males); or “ex-smoker” (smoking cessation ≥2 years; 18 males).

### 2.2. Serum Isolation

Blood samples were collected without anticoagulants in a vacuum-sealed blood collection tube, and left undisturbed at room temperature for 20 min to allow them to clot (the participant had fasted after 5 p.m. the previous day). The clots were then removed by centrifuging the samples at 1500 g for 10 min in a refrigerated centrifuge. The resulting supernatant was considered serum. Following centrifugation, the serum was immediately transferred to a clean polypropylene tube using a Pasteur pipette. The samples were maintained at 2–8 °C during handling and were immediately analyzed, avoiding the freeze-thaw cycle that could harm certain serum components.

### 2.3. Assessment of Inflammatory Markers

Seventeen cytokines and chemokines (IL-α, IL-1β, IL-6, IL-8, CXCL9/MIG, sIL-6R, SAA, BCA-1, CRP, TARC, MDC, TNFRII, ITAC, SCF, ENA-78, sVEGFR3, and TNF-α) from the serum samples were analyzed using the Bio-Plex 200 system (Bio-Rad, Hercules, CA, USA) according to the manufacturer’s instructions, and the lower limits of detection (LOD) for these cytokines and chemokines were 81.8, 0.4, 2.3, 1.9, 1.8, 257.6, 1100, 0.7, 4, 1.7, 0.9, 30.3, 0.1, 1.5, 7.3, 18.01, and 0.9 pg/mL, respectively. The magnetic bead-based multiplex immunoassay principle is based on the sandwich enzyme-linked immunosorbent assay (ELISA) model, where magnetic beads are covalently bound to antibodies that react to the targeted biomarker. Measurements were performed twice, in accordance with the manufacturer’s instructions. The human serum samples were stored at 2–8 °C, and determined within 4 h. The cytokine assay plates were wet with an assay buffer and washed twice with a wash buffer. Two magnetic beads were added to the 96-well plate and serial dilutions of the reconstituted standard and samples. Second detection antibodies with streptavidin–phycoerythrin conjugate completed the sandwich complex. Data from the reactions were acquired using the Bio-Plex200 reader, a digital processor managed data output, and the Bio-Plex Manager software generated data as Median Fluorescence Intensity (MFI) and concentration (pg/mL).

### 2.4. Statistical Analysis

Data were analyzed for each set of experiments, by calculating medians and inter-quartile ranges (IQRs). Scatter plots were used to show the values of the median, IQR, and Tukey whiskers. The nonparametric Kruskal–Wallis test, and Dunn’s multiple comparison test, were used when appropriate. Probability (*p*) values were calculated, based on two-tailed tests. Data analysis was performed using GraphPad Prism software version 5.0 (GraphPad Software, Inc., La Jolla, CA, USA). A *p*-value of less than 0.05 was considered statistically significant.

## 3. Results

The study included 78 subjects with a mean age of 37.5 years. These included non-smokers (30 males), current smokers (30 males), and ex-smokers (18 males). The characteristics of gender and smoking status of the study population are shown in Table 1: 

A total of 17 cytokines and chemokines were tested in 78 samples, and were roughly divided into four groups based on their expression across the three groups. CXCL9/MIG and sIL-6R were continuously up-regulated in the three groups, and TARC, ITAC, sVEGFR3, and serum amyloid A protein (SAA) returned to normal levels after smoking cessation. IL-1β, BCA-1, tumor necrosis factor-α (TNF-α), CRP, ENA-78, MDC, and TNFRII did not significantly differ across the groups. The production of these molecules was constant. Several cytokines (IL-1α, IL-6, IL-8, and SCF) were below the lower LOD in all subjects, either because levels were very low or because the molecules were not produced by healthy subjects. The expression levels of various cytokines and chemokines in different groups were shown in Table 2: 

Values are expressed as pg/mL, median (IQR); differences between groups were not statistically significant (*p* > 0.05) after performing a Kruskal–Wallis test and Dunn’s multiple comparison test. The left side of the *p*-value column indicates the result of the differential analysis between non-smokers and current-smokers, and the right side is the result of the differential analysis between current-smokers and ex-smokers. Cytokines and chemokines were not detected in any group, either because they were under the lower limit of detection (LOD, pg/mL) or because they were not produced.

### 3.1. Cytokine Levels Significantly Increased in Non-Smokers, Current Smokers, and Ex-Smokers

**Figure 1:**
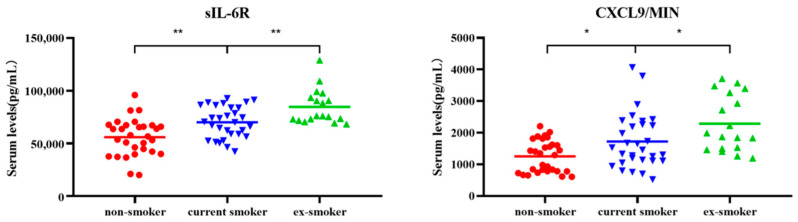
Scatter plots of serum levels (pg/mL) of cytokines CXCL9/MIG and sIL-6R for non-smokers (*n* = 30), current smokers (*n* = 30), and ex-smokers (*n* = 18). Whiskers were calculated using the Tukey method. Outliers not shown. * *p* < 0.05, ** *p* < 0.01: Kruskal–Wallis test and Dunn’s multiple comparison test.

Levels of CXCL9/MIG and sIL-6R in the non-smoker group were significantly lower when compared to the current smoker and ex-smoker groups. Additionally, the levels of both CXCL9/MIG and interleukin 6 soluble receptor (sIL-6R) gradually increased in the non-smokers (*n* = 30), current smokers (*n* = 30), and ex-smokers (*n* = 18) (Figure 1).

### 3.2. Changes in Four Types of Cytokines

**Figure 2:**
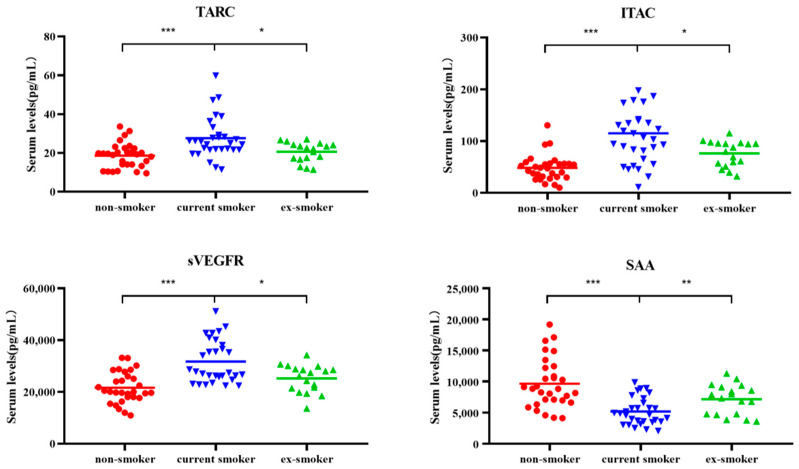
Scatter plots of serum levels (pg/mL) of cytokines TARC, ITAC, sVEGFR3, and SAA for non-smokers (*n* = 30), current smokers (*n* = 30), and ex-smokers (*n* = 18). Whiskers calculated using the Tukey method. Outliers not shown. * *p* < 0.05, ** *p* < 0.01, *** *p* < 0.001: Kruskal–Wallis test and Dunn’s multiple comparison test.

The levels of human TARC, ITAC, and sVEGFR3, were all significantly higher in current smokers than in non-smokers and ex-smokers. However, SAA content was lowest in current smokers and highest in ex-smokers (Figure 2).

## 4. Discussion

This study assessed the effects of smoking on the inflammatory cytokine levels of 78 adult male subjects, identifying substantial differences in several immune cytokines between non-smokers (*n* = 30), current smokers (*n* = 30) and ex-smokers (*n* = 18). These cytokines mediate various mechanisms of the immune and inflammation responses, such as the chemotaxis of T cells and eosinophils, inflammation, and anti-inflammation processes. In addition, cytokines are also involved in cell development/differentiation, cell growth and activation, angiogenesis, and hematopoiesis. These findings provide strong evidence that smoking could affect systemic immunity and inflammation.

Toxicants in cigarette smoke can alter several pulmonary and systemic immune characteristics related to several immune cells, including increases in macrophages, neutrophils, eosinophils, and mast cells, and the functionality of various immune cells [46]. Compared to the non-smokers, the expression of CXCL9/MIG and sIL-6R in the serum of the current smokers and ex-smokers continued to increase. CXCL9/MIG serves as an important antiviral defense, and plays an important role in the development or prevention of certain lung diseases [47,48,49]. CXCL9/MIG is one of three chemokines, along with CXCL10 and CXCL11, that are highly induced by IFN-γ, as well as by type I and III IFNs [48,50,51]. Our results demonstrate that smoking increases cytokine CXCL9/MIG expression, which can partially explain the pro-inflammatory and pro-atherosclerotic properties of smoking, and could be one of the adverse side effects of long-term nicotine use. In the blood of healthy human body, sIL-6R can be detected [52], and sIL-6R expression could increase in some immune diseases, such as peritonitis [53] and rheumatoid arthritis [54]. This suggests that sIL-6R play an important role in immune diseases. Studies have reported the immune effects of IL-6 and sIL-6R in HBV-infected humans. Increased serum sIL-6R concentrations have been observed in patients with hepatitis B treated with interferon [55]. The concentration of serum sIL-6R did not increase in patients who experienced no effects after interferon treatment. Studies have demonstrated that smoking status is closely related to increases in sIL-6R receptor levels in human serum [56], which is consistent with the results of this study. In the former smoker group, the expression of sIL-6R continued to increase, which could be due to overcompensation caused by metabolic adaptation after smoking cessation [56]. In this study, we found that the levels of CXCL9/MIG and sIL-6R in serum increased after smoking, while the levels of these two cytokines did not decrease after smoking cessation. These results indicate that smoking could exacerbate inflammation, and that inflammation did not decrease or increase continuously after quitting smoking. This suggests that continuous smoking could increase some inflammatory factors. At the same time, the body adapted, and quitting smoking disrupted this stability, further increasing relevant inflammatory factors. It remains unclear whether inflammatory responses decrease after smoking cessation or how long the inflammatory response would decrease after smoking cessation for the body to re-adapt.

Our results demonstrated that there were significant differences in inflammatory cell molecules after quitting smoking. Among ex-smokers, the levels of chemokines (TARC, ITAC) and growth factor receptors (sVEGFR3) approached levels observed in non-smokers, as the time since smoking cessation increased. Therefore, smoking could change those cytokines that gradually revert to normal levels once an individual stops smoking. It has been reported that concentrations of TARC in the serum decrease once an individual quits smoking, and are associated with a decrease in pulmonary inflammation [57]. In addition, the levels of ITAC and sVEGFR3 in ex-smokers were significantly lower than the levels in the smoker group, which was consistent with the results of Sami, et al., [58,59]. ITAC is related to triggering an inflammatory response and inhibiting the growth of vascular endothelial cells [36]. sVEGFR3 regulates lymphangiogenesis by inhibiting VEGF-C, and preventing it from activating its homologous receptor. Overexpression of sVEGFR3 in lung cancer cells can effectively inhibit the density of lymphatic vessels in the tumor, and reduce the incidence of lymph node metastasis [60]. Smoking can damage microvascular function and vascular endothelial function, and reduce the synthesis of oxyhemoglobin [61]. The decreased expression of ITAC after smoking cessation could be related to reduced inflammation and/or the normal growth of vascular endothelial cells [36]. sVEGFR-3 is a novel soluble vascular endothelial growth factor receptor [37], and it is a potential biomarker for the antiangiogenic activity of tyrosine kinase inhibitors [38]. Gene therapy for endometrial cancer lymph node and lung metastases is made possible using muscle-mediated sVEGFR-3 expression [39]. This study demonstrates that the expression levels of ITAC and sVEGFR3 increased in current smokers, and that these expression levels significantly decreased after smoking cessation, which could be because smoking triggers an inflammatory response that leads to vascular endothelial dysfunction and a certain degree of recovery after smoking cessation.

Our findings suggest that current smokers may have suppressed levels of systemic immune markers, with reduced systemic levels of certain immune/inflammatory cytokines (SAAs), compared to non-smokers, and increased levels after smoking cessation, and that the SAA level of current smokers significantly differed from that of non-smokers and ex-smokers. This was different from the finding that there was no difference in SAA levels before and after smoking [62]. However, this could be due to the small sample size (10 normal smokers and 10 moderate smokers with persistent asthma) and individual differences. Our findings also differed from the findings that SAA low-density lipoprotein levels showed a significant upward trend from non-smokers to current smokers [63]. SAA is pro-inflammatory because it is a potent chemokine that mediates leukocyte migration and can also stimulate the expression of pro-inflammatory mediators under in vitro and in vivo conditions [40,41,42]. Systemic inflammation is common in COPD and lung cancer, and SAA is predictive of severity in both diseases, but the subject group was 578 obese Japanese outpatients [63]. Therefore, the different results obtained by different studies might be related to the sample size, race, health status, age, and other factors of the test group, which requires further discussion and research.

Four kinds of cytokines were not detected in the serum of all healthy subjects in this study. Some of these molecules are unique to certain pathological situations or are related to an inflammatory response. For example, IL-1α, IL-6, and IL-8 are all molecules with a strong inflammatory role [21,64,65,66]. Previous studies have found that levels of IL-6, IL-1 α, and IL-8 in smokers were higher than those in ex-smokers because exposure to cigarette smoke increases oxidative stress [18,23]. We did not detect IL-6, IL-1 α, or IL-8 in our study, which could be due to the sample size. In addition, previous studies have found SCF to be elevated in pancreatic ductal adenocarcinoma patients, and SCF plays an important role in the pathophysiology of mast cells [67,68]. Other cytokines (IL-1β, BCA-1, TNF-α, CRP, ENA-78, MDC, and TNFRII) showed no obvious differences between the non-smoker (*n* = 30), current smoker (*n* = 30), and former smoker groups (*n* = 18). This suggests that smoking has no obvious effect on the expression pathways of MDC and TNFRII.

We analyzed the serum collected from the three types of subjects, so the levels we detected were those of mature and circulating cytokines and chemokines. We did not analyze the relationship between the number of cigarettes smoked and the inflammatory factor levels. However, this study could be useful as a reference to help evaluate the serum levels of cytokines and chemokines in healthy subjects of different smoking statuses. Our results suggest that inflammatory cytokines such as CXCL9/MIG, sIL-6R, and TARC could add moderate predictive value when evaluating smoking-related lung diseases. Additional studies, ideally with larger sample sizes and more diverse subject groups, are needed to better understand how these cytokines and chemokines are produced and modified under different environmental and physiological conditions.

## 5. Conclusions

In this study, serum samples from 78 healthy male volunteers (aged 18–60) were analyzed using the suspension chip technique to measure 17 different inflammatory and immune cytokines related to three smoking statuses. Long-term smoking can result in the up-regulation of CXCL9/MIG and sIL-6R, a trend that continues to increase after quitting. Smoking caused the levels of TARC, ITAC, and sVEGFR3 to be up-regulated. After quitting, these levels gradually decreased, and almost declined to levels observed in the non-smokers. This study found that smoking had a decreasing effect on SAA, but levels of SAA expression increased to that of non-smokers after smoking cessation. Smoking does not affect the expression of MDC, CRP, IL-1β, BCA-1, TGF-a, ENA-78, or TNFRII, which could require a larger sample size for a more refined analysis. Assessing the levels of IL-1α, IL-6, IL-8, and SCF will also require a larger sample size along with a more sensitive detection method. Therefore, smoking or smoking cessation will cause significant changes in cytokine levels. Changes in related cytokines after smoking cessation are mainly restorative, while some cytokines further strengthen the trend of smoking-related changes.

## Figures and Tables

**Table 1:** Characteristics of the subject sample (*n* = 78).

Group	Frequency	Age (Median; Min/Max)	Sex	Race
non-smokers	30	36.6 yr; 19/57 yr	Male	Han
current-smokers	30	35.5 yr; 24/49 yr	Male	Han
ex-smokers	18	42.2 yr; 26/56 yr	Male	Han
Total	78	37.5 yr; 19/57 yr	Male	Han

**Table 2:** Expression of cytokines and chemokines in three groups (pg/mL).

Expression Trends	Cytokine	Non-Smokers	Current-Smokers	Ex-Smokers	*p*-Value
Continuous increase	CXCL9/MIG	1320.65	1493.70	1928.06	0.03/0.03
	sIL-6R	57,248.93	70,286.72	76,155.75	2.96 × 10^−3^/6.84 × 10^−3^
Recovery after smoking cessation	TARC	19.47	25.47	22.39	1.28 × 10^−4^/1.61 × 10^−2^
	ITAC	48.09	105.34	84.09	0.07 × 10^−4^/3.89 × 10^−2^
	sVEGFR3	20,058.64	27,610.30	26,732.49	0.06 × 10^−4^/2.53 × 10^−2^
	SAA	8833.60	4771.36	7609.59	0.03 × 10^−4^/9.37 × 10^−3^
No significant difference	IL-1β	12.35	22.21	38.24	0.35/0.43
	BCA-1	24.92	39.37	27.55	0.84/0.35
	TNF-α	11.21	13.95	10.63	0.99/0.191
	CRP	9055.50	7058.28	11,859.6	0.918/0.30
	ENA-78	547.60	457.60	649.28	0.51/0.34
	MDC	408.08	430.63	396.81	0.47/0.34
	TNFRII	14,775.96	15,543.39	13,188.03	0.94/0.17
Not detected	IL-1α	—	—	—	/
	IL-6	—	—	—	/
	IL-8	—	—	—	/
	SCF	—	—	—	/

## Data Availability

Not applicable.

## Sample Availability

Samples of some compounds are available from the authors.
